# Dexamethasone inhibits brain apoptosis in mice with eosinophilic meningitis caused by *Angiostrongylus cantonensis* infection

**DOI:** 10.1186/s13071-015-0792-7

**Published:** 2015-04-02

**Authors:** Hung-Chin Tsai, Bi-Yao Lee, Chuan-Min Yen, Shue-Ren Wann, Susan Shin-Jung Lee, Yao-Shen Chen

**Affiliations:** Section of Infectious Diseases, Department of Medicine, Kaohsiung Veterans General Hospital, Kaohsiung, Taiwan and National Yang-Ming University, Taipei, Taiwan; Department of Parasitology and Graduate Institute of Medicine, Kaohsiung Medical University, Kaohsiung, Taiwan; National Defense Medical Center, Taipei, Taiwan

**Keywords:** Angiostrongylus cantonensis, Apoptosis, Blood brain barrier, Corticosteroid, Eosinophilic meningitis

## Abstract

**Background:**

*Angiostrongylus cantonensis*, the rat lungworm, is the major cause of eosinophilic meningitis worldwide. Rats serve as the definitive host of the nematode, but humans can be infected incidentally, leading to eosinophilic meningitis. A previous BALB/c animal study has demonstrated increased apoptotic proteins and decreased anti-apoptotic proteins in mice infected with *A. cantonensis*. Steroids may be an effective treatment option for eosinophilic meningitis caused by *A. cantonensis*, but the involved mechanism is unclear. This study hypothesized that the beneficial effects of steroids on eosinophilic meningitis are mediated by decreased apoptosis.

**Methods:**

In a BALB/c animal model, mice were orally infected with 50 *A. cantonensis* L3 via an oro-gastric tube and were sacrificed every week for 3 consecutive weeks after infection or until the end of the study. Dexamethasone was injected intra-peritoneally from the 7^th^ day post-infection until the end of the 21-day study. Evans blue method was used to measure changes in the blood brain barrier, while western blotting, immuno-histochemistry, and TUNEL assay were used to analyze brain homogenates expression of apoptotic and anti-apoptotic proteins.

**Results:**

There were increased amounts of Evans blue, apoptotic proteins (caspase-3, -8, and -9 and cytochrome C), and decreased anti-apoptotic proteins (bcl-2) after 2-3 weeks of infection. Dexamethasone administration significantly decreased Evans blue extravasations and apoptotic protein expressions.

**Conclusions:**

Apoptosis of mice brain homogenates can be repressed by dexamethasone treatment.

## Background

*Angiostrongylus cantonensis,* also known as rat lungworm, is the most common cause of eosinophilic meningitis worldwide. This parasitic infection is endemic in the Southeast Asian and Pacific regions [1-4] and the intermediate hosts are fish and snails [5-8]. The typical clinical presentation is acute meningitis, with an eosinophilic pleocytosis. The pathological findings in the central nervous system (CNS) include meningitis with a predominance of eosinophils and plasma cells; tortuous tracks of various sizes in the brain and spinal cord surrounded by an inflammatory reaction and degenerating neurons; granulomatous response to the dead parasites; and non-specific vascular reactions, including thrombosis, vessel rupture, arteritis, and aneurysm formation [9]. However, changes in inflammatory cytokines and specific antibody to *A. cantonensis* after infection, as well as the mechanism of blood brain barrier damage, remain unclear.

A previous study has shown that eosinophilic meningitis caused by *A. cantonensis* in infected mice, with activation of the apoptosis pathway in leukocytes infiltrating the subarachnoid space [10]. Steroids have been proposed as adjunct treatment [11,12], but how the apoptosis process is regulated in the brain parenchyma and roles of steroid administration are also unknown.

Using a BALB/c mice model, this study hypothesized that the treatment effect of steroids in eosinophilic meningitis is mediated by the down-regulation of apoptosis.

## Methods

### Ethics statement

The animal studies were conducted in strict accordance with the recommendations from Taiwan’s Animal Protection Act. The Animal Committee of Kaohsiung Veterans General Hospital approved the study protocol.

### Infection of BALB/c mice and intra-peritoneal steroid injection

Forty BALB/c mice aged 6-7 weeks were purchased from the National Laboratory Animal Breeding Research Centre. They were raised and maintained in an air-conditioned animal facility (25 ± 2°C and 50 ± 10% relative humidity). Third-stage larvae of *A. cantonensis* were harvested from infected *Biomphalaria glabrata* after treatment with artificial gastric juice (pepsin, 2 g; concentrated HCl, 7 mL; and distilled water, 1 L) as previously described [13]. The mice were orally infected with 50 *A. cantonensis* L3 via an orogastric tube after slight ether anesthesia. Eight mice were sacrificed every week for 3 consecutive weeks after infection (end of the study). Dexamethasone (500 ug/kg/day) was injected intra-peritoneally from 7^th^ post-infection day until the end of the study. All animal experiments were approved by the Institutional Animal Care and Use Committee.

### Measurement of permeability of the blood–brain barrier by Evans blue method

A volume of 200 μL of 2% (w/v) solution of Evans blue in PBS was injected into the tail vein of each mouse. The mouse’s brain was removed one hour later, after anesthesia with ketamine, which was ground with 1.0 mL PBS in a glass-tissue grinder using a Teflon pestle. The extract was then centrifuged at 18,000 × *g* (Hermle, Z326K, Germany) for 10 min at room temperature. The optical density of the supernatant was read at 595 nm wavelength using a colorimeter (Thermo Scientific Multiskan FC, USA).

### Western blotting

The mouse brain extracts were centrifuged at 12,000g for 20 min in 4°C to remove the debris. Protein concentration was analyzed using a protein assay kit (Bio-Rad, Hercules, CA, USA) with BSA as the standard. Briefly, 50μg protein were separated by 10% SDS-PAGE and transferred to PVDF membranes. The membranes were incubated in blocking buffer for 60 min (PBS containing 5% BSA) at room temperature. The membranes were then washed three times with PBST and probed with caspase-3, -8, and -9 and Bcl-2 and cytochrome C with β-actin (Sigma, St. Louis, MO, USA) as control. After incubation with secondary antibody, detection was made by enhanced chemi-luminescence method. Quantitative analysis of the bands was performed using a computer-assisted imaging densitometer system, UN-SCAN-IT™.

### Immuno-histochemistry

Immuno-histochemistry was performed to detect apoptotic proteins on brain sections. Briefly, paraffin-embedded sections (4 μm) were de-paraffinized, treated with 3% H_2_O_2_ in methanol for 10 min to inactivate any endogenous peroxidase, and washed with PBST (0.1% Tween 20 in PBS). The sections were then blocked for 60 min in blocking buffer (10% bovine serum albumin [BSA] in PBS), followed by a 60 min incubation with rabbit polyclonal IgG and rabbit monoclonal IgG in 1% BSA. The sections were each washed three times for 10 min in PBST, followed by 60 min incubation with HRP-conjugated secondary antibody (Novolink™ polymer detection kits) at room temperature. The sections were then incubated for 10 sec-1 min at room temperature with 3,3’-diaminobenzidine (DAB) for color development, and then counterstained with diluted hematoxylin.

### Terminal deoxynucleotidyltransferase-mediated dUTP nick-end labeling (TUNEL) assay

Cells undergoing apoptosis generate free DNA ends that were labeled *in situ* using terminal deoxynucleotidyltransferase (TdT) by incorporating an exogenously added, labeled nucleotide into the DNA strand. *In situ* Death Detection Kits (Roche, Mannheim, Germany) detect apoptotic cells by specific staining. TUNEL assay was used to determine brain cell apoptosis. Briefly, brain sections on cover slips were de-paraffinized and washed with PBS. Sections were treated with 3% H_2_O_2_ in methanol for 10 min to inactivate endogenous peroxidase and washed three times with PBS for 5 min. The DNA fragments were labeled with the TUNEL mix fluorescence for 1 hour than washed three times with PBS for 5 min. The sections were incubated in DAPI for 10 min and observed by fluorescence microscopy.

### Statistical analysis

The Mann Whitney *U* test was used to compare changes in apoptotic and anti-apoptotic proteins, and in Evans blue amounts every week in relation to controls or steroid treatment. Statistical significance was set at *p* < 0.05.

## Results

### Permeability of the blood-brain barrier by Evans blue method

**Figure 1:**
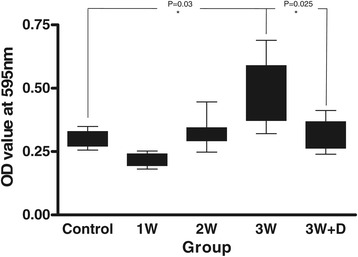
**The amount of Evans blue staining in the mice brain significantly increased at 3 weeks post-infection compared to those of uninfected mice (** ***p*** **= 0.03).** Dexamethasone administration for 2 weeks (7^th^–21^st^ day post-infection, group 3W + D) significantly decreased the intensity of Evans blue staining compared to those of mice at 3 weeks post-infection, which had the most serious damage to the blood-brain barrier (p = 0.025).

After infection, the brains of infected mice showed varying degrees of staining by Evans blue. The quantities of Evans blue in the homogenates of the brain gradually increased 14 days post-infection until the end of this study (21 days post-infection). The amount of Evans blue in the mice brain showed significant increase 3 weeks after infection compared to those of uninfected mice (*p* < 0.05). Administration of dexamethasone for 2 weeks significantly decreased the intensity of Evans blue in the brain of mice compared to those of untreated mice 3 weeks after infection (Figure 1).

### Western blot analysis of apoptotic protein concentrations in the brain homogenates of mice

**Figure 2:**
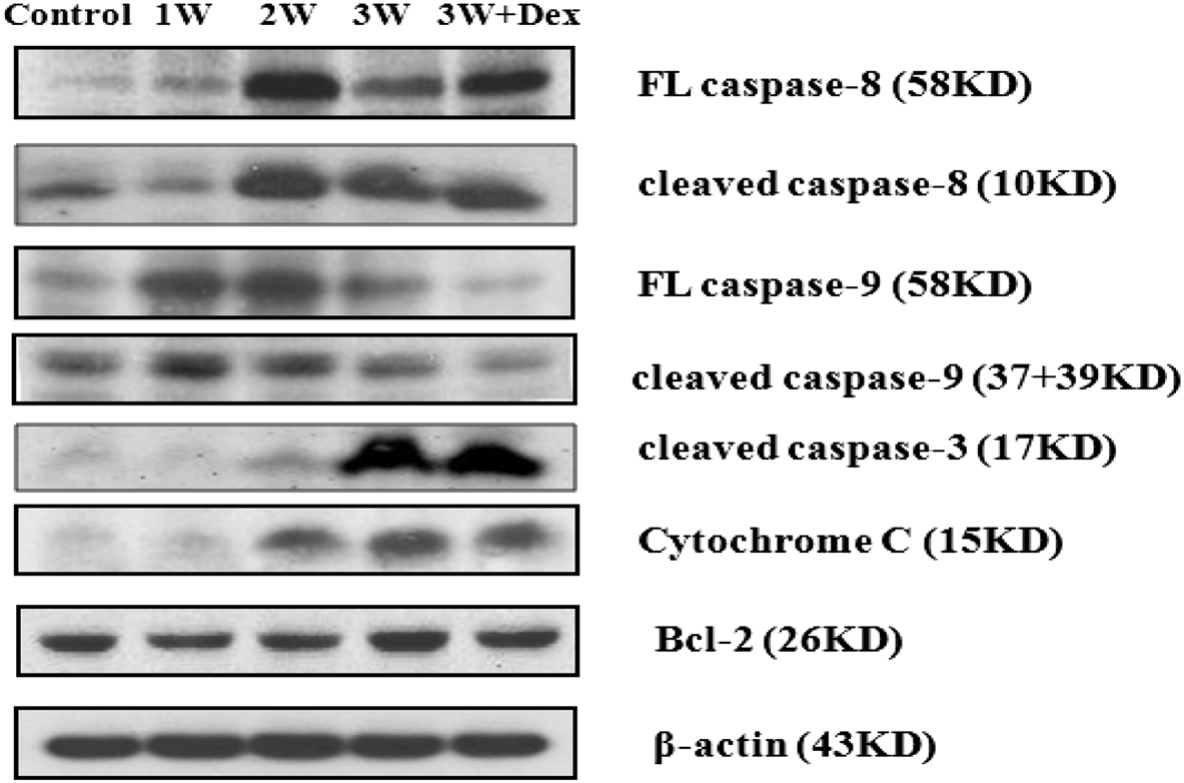
**By Western blotting, there were significant increases in caspase-3, -8, and -9 and in cytochrome C proteins in the 2** ^**nd**^ **and 3** ^**rd**^ **weeks post-infection compared to the controls and on the 1** ^**st**^ **week post-infection.** Dexamethasone administration significantly decreased the expression of cleaved caspase-3, -8, and -9 and cytochrome C proteins. Control: no parasitic infection; 1W: one week after infection; 2W: two weeks after infection; 3W: three weeks after infection; 3W + Dex: mice given dexamethasone for 2 weeks (7^th^–21^st^ day post-infection) and sacrificed on day 21.

Western blot analysis for apoptotic protein in brain homogenates (Figure 2) revealed that apoptotic proteins caspase-8, -3, and -9 and cytochrome C increased post-infection. There was also a significant increase in caspase-3 and -8 and in cytochrome C protein 2-3 weeks post-infection compared to the controls and levels at 1 week post-infection. Dexamethasone administration significantly decreased caspase-9 and cytochrome C protein expressions. However, there was no effect on the expression of Bcl-2 protein compared to levels within the first three weeks post-infection.

### Immuno-histochemistry (IHC) staining of apoptotic protein in the meninges of mice brain

**Figure 3:**
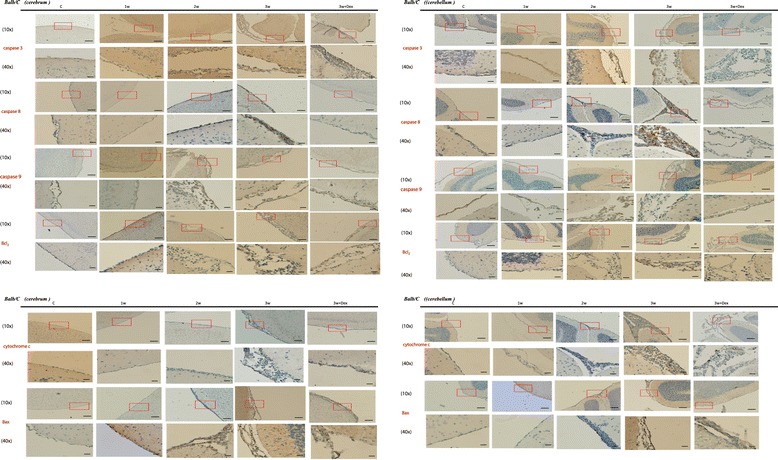
**By immuno-histochemistry (IHC), there was an increase in apoptotic protein expressions in the brain meninges on the 2** ^**nd**^ **and 3** ^**rd**^ **weeks post-infection compared to the controls and on the 1** ^**st**^ **week post-infection in both the cerebrum and cerebellum.** Dexamethasone administration significantly decreased the expressions of apoptotic proteins. Scale bar, 10 um at 10x and 40x magnification.

The IHC study for apoptotic protein in brain meninges (Figure 3) revealed an increase of apoptotic protein amounts in the 2^nd^ and 3^rd^ week post-infection compared to the controls and at 1 week post-infection in both the cerebrum and cerebellum. Dexamethasone administration significantly decreased apoptotic protein expressions. However, the decrease in expressions of apoptotic proteins in the brain meninges was specific and not every protein had a similar decrease after steroid administration.

### TUNEL assay in the subarachnoid space and meningeal area of mice infected with A. cantonensis

**Figure 4:**
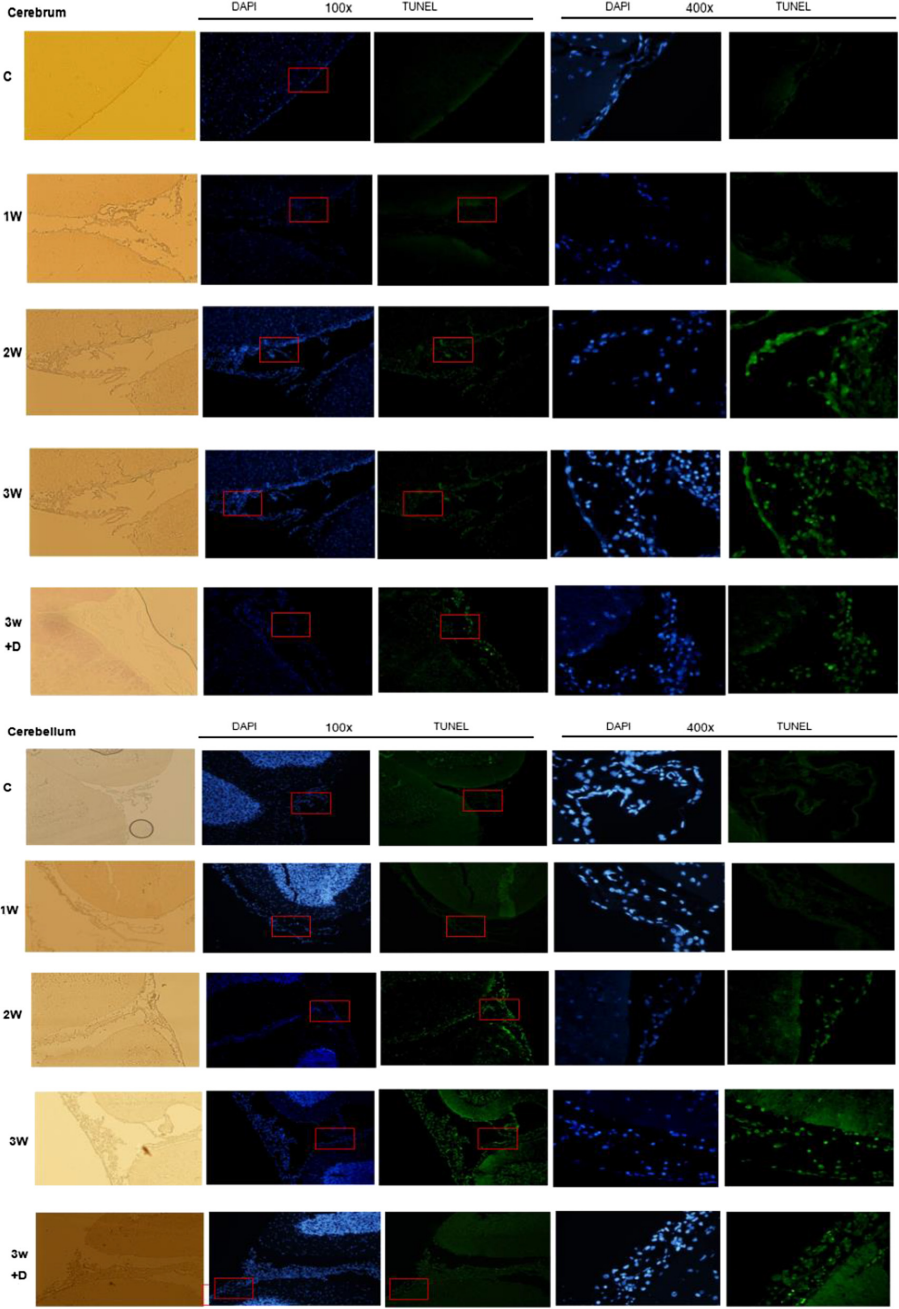
**TUNEL assays of brain apoptosis in mice infected with** ***A. cantonensis*** **revealed increased apoptosis 3 weeks post-infection.** Steroids significantly decreased this apoptosis.

Brain tissues were harvested from BALB/c mice 1-3 weeks after infection and dexamethasone was given for 2 weeks post-infection, with third stage larvae of *A. cantonensis*, and then analyzed by TUNEL staining at different weeks of infection. Immuno-fluorescence TUNEL (green) and DAPI (blue) were used to stain the apoptotic cells. In the cerebrum and cerebellum, TUNEL stain was near maximal on the 3^rd^ week post-infection. Dexamethasone administration decreased cell apoptosis compared to the first three weeks infection (Figure 4).

## Discussion

This study reveals an increase in brain apoptotic protein expressions and blood-brain barrier damage as demonstrated by increase Evans blue extravasations following 2-3 weeks of infection with third stage larvae of *A. cantonensis*. Steroid administration remarkably decreases the Evans Blue staining and apoptotic protein expressions. These findings provide evidences supporting the effects of steroids on *A. cantonensis* infection.

Steroids have been proposed as adjunctive treatment for eosinophilic meningitis. In the clinical observations of Chotmongkol et al. [11], a two-week course of prednisolone is beneficial in relieving headaches in patients with eosinophilic meningitis. In the study of Sawanyawisuth et al. [12], a one-week course of corticosteroid treatment is also effective. Steroids may improve capillary function by reducing the activity of urokinase-type plasminogen activators and matrix metalloproteinase 9, and increasing the levels of matrix metalloproteinase tissue inhibitors [14]. The use of dexamethasone in mice with eosinophilic meningitis caused by *A. cantonensis* infection can partially inhibit the activity of plasminogen activators and inflammation [15].

Treatment of eosinophilic meningitis with corticosteroids can also relieve intracranial pressure and improve neurologic symptoms due to inflammatory responses to migrating and dying worms [16]. IL-5 can regulate the proliferation, differentiation, and activation of eosinophils [17] in parasitic meningitis, and provide a signal for the mobilization of eosinophils from the bone marrow [18]. Steroids can suppress the inflammatory gene transcriptions of interleukin-3, interleukin-4, GM-CSF, and various chemokines, thereby decreasing the survival of eosinophils [19]. Taken together, the beneficial effects of steroids on eosinophilic meningitis caused by *A. cantonensis* infection are possibly mediated by the down-regulation of apoptotic protein expressions in the CSF, improvement of blood-brain barrier dysfunction, inhibition of the activity of plasminogen activators and inflammation, and the inhibition of cytokine-dependent eosinophil survival, among others.

The term apoptosis was first coined in 1972 and has since been generally applied to programmed cell death, where cells die by an active process characterized by nuclear fragmentation and cell shrinkage, while maintaining cell membrane integrity [20]. Apoptosis is characterized by two major pathways. One is called the “extrinsic” pathway, triggered by ligation of trans-membrane death receptors belonging to the tumor necrosis factor (TNF) receptor super-family [21]. The other one is the “intrinsic” pathway, mainly regulated by the interplay of pro- and anti-apoptotic members of the B cell lymphoma 2 (Bcl-2) family at the level of the mitochondria [22]. This is initiated by cytotoxic drugs, intracellular stress, growth-factor deprivation, and hormones that cause the activation of pro-apoptotic Bcl-2 family members [23] and subsequently, the release of cytochrome c from the mitochondria. Together with Apaf-1 and procaspase-9, this results in the formation of the “apoptosome”, a multimeric complex that activates caspase-3.

The interaction between pro- and anti-apoptosis factors in the brain has important regulatory functions in the patho-physiologic process, including the development of neuronal ischemic infarction after both global and focal ischemia [24-26]. In chronic neurodegenerative diseases like Alzheimer’s disease, amyotrophic lateral sclerosis, Parkinson’s disease, and Huntington’s disease, and in acute or sub-acute pathologic conditions such as ischemia, toxins, trauma and infection, apoptosis causes significant neuronal loss through these processes [27-29].

Apoptosis is also essential for the growth and survival of most multi-cellular organisms [30]. In a mice model of parasitic eosinophilic meningitis caused by *A. cantonensis* infection, Chen et al. have identified increased infiltrations of leukocytes in the brain parenchyma and in the subarachnoid space, as well as apoptosis 15-25 days after mice are infected with third stage larvae of *A. cantonensis* [10]. In the study of Hu et al., the larvae extracts of *A. cantonensis* can induce apoptosis in primary culture brain micro-vascular endothelial cells and in brain astrocytic cells, and participate in blood-brain barrier dysfunction [31]. Chuang et al. used ICR mice as a parasitic eosinophilic meningitis model to show that apoptosis of inflammatory cells can be induced when the infected mice are treated with Mebendazole and/or IL-12 [32]. The model of steroid-induced cell death assumes that various pro- and anti-apoptotic Bcl-2 family members are implicated in steroid induced apoptosis [33].

The over-expression of the anti-apoptotic family members Bcl-2 or Bcl-XL prevents steroid-induced apoptosis, whereas the combined disruption of the pro-apoptotic proteins Bax and Bak results in complete resistance to steroid-induced cell death [34]. Several caspases have been proposed to be necessary for apoptosis induction by steroids. Data obtained in the MOG-EAE model in C57Bl ⁄ 6 mice further suggest that steroids primarily induce apoptosis in peripheral T cells, whereas cell death in the CNS appears to be a secondary event [35].

In the present study, dexamethasone is associated with the inhibition of apoptosis instead of the induction of apoptosis. This discrepancy may be due to several reasons. First, this study was *in vivo* and previous studies were mostly *in vitro* cell model. Second, different strains of mouse (BALB/c vs. C57BL/6) were used. Third, different disease models for steroid use were also involved here. Lastly, the dosage, routine of administration, and duration (2 weeks) were different in different disease models.

## Conclusion

Apoptosis of mice brain homogenates can be repressed by treatment with dexamethasone. The findings here demonstrate one mechanism of action of corticosteroids in the treatment of *A. cantonensis* eosinophilic meningitis.
